# The curious case of vanishing mitochondria

**DOI:** 10.15698/mic2016.10.531

**Published:** 2016-09-30

**Authors:** Anna Karnkowska, Vladimír Hampl

**Affiliations:** 1 Department of Botany, University of British Columbia, Vancouver, British Columbia, Canada V6T 1Z4.; 2 Department of Parasitology, Charles University, Prague, Czech Republic.

**Keywords:** amitochondriate, iron-sulphur cluster synthesis, mitochondrion, mitochondrion-related organelles, Monocercomonoides sp

## Abstract

Due to their involvement in the energy metabolism, mitochondria are essential for most eukaryotic cells. Microbial eukaryotes living in low oxygen environments possess reduced forms of mitochondria, namely mitochondrion-related organelles (MROs). These do not produce ATP by oxidative phosphorylation on their membranes and some do not produce ATP at all. Still, they are indispensable because of other essential functions such as iron-sulphur (Fe-S) cluster assembly. Recently, the first microbial eukaryote with neither mitochondrion nor MRO was characterized - *Monocercomonoides *sp. Genome and transcriptome sequencing of *Monocercomonoides* revealed that it lacks all hallmark mitochondrial proteins. Crucially, the essential mitochondrial pathway for the Fe-S cluster assembly (ISC) was replaced by a bacterial sulphur mobilization (SUF) system. The discovery of such *bona fide *amitochondriate eukaryote broadens our knowledge about the diversity and plasticity of eukaryotic cells and provides a substantial contribution to our understanding of eukaryotic cell evolution.

## INTRODUCTION

The endosymbiotic origin of the mitochondrion from an alpha-proteobacterium is crucial for the understanding of eukaryogenesis. Whether it happened early or late in the evolution of eukaryotes is still heatedly debated 1, yet it is fairly certain that all extant eukaryotes known to science evolved from a mitochondriate common ancestor. The process of the organelle establishment was rather complicated and is not well understood. During the transition from a bacterial symbiont to a proto-organelle, 1000-3000 genes were lost or transferred to the nuclear genome of the host 2. Only small fractions of the current mitochondrial proteomes are encoded in the respective mitochondrial genomes, while the majority of the proteins are encoded in nuclei and targeted to mitochondria. The targeting system relies on a targeting signal and an import process, which involves translocases of the outer membrane (TOM) and inner membrane (TIM), a sorting and assembly machinery (SAM) and mitochondrial chaperones. This protein import machinery is one of the hallmarks of mitochondria, and it is conserved to a certain degree among all eukaryotes, suggesting its single common origin.

## REDUCED FORMS OF MITOCHONDRIA

Since the time when Lynn Margulis proposed the serial endosymbiotic theory (SET) for the origin of eukaryotes and mitochondria 3, our view on this key evolutionary event has progressed. One of the interesting assumptions of SET and the follow-up Archezoa hypothesis 4 is that primitively amitochondrial eukaryotes (Archezoa) existed before the mitochondrial endosymbiosis, existed in the past and some of their descending lineages, which did not pass through mitochondrial endosymbiosis, may still live on Earth today. This inference was supported by the studies on anaerobic or microaerophilic microbial eukaryotes, like *Giardia*, *Trichomonas*, *Entamoeba* or microsporidia. They all seemed to lack mitochondria and they grouped together at the base of the phylogenetic trees constructed using SSU sequences, which made them ideal candidates for lineages of Archezoa. An important turning point of this story was the discovery of MROs in all these ‘Archezoans’. In 1995 Clark and Roger demonstrated that *Entamoeba histolytica* contains genes encoding proteins that in all other eukaryotes are localized in the mitochondrion 5. Since then, many similar studies have shown the presence of genes encoding mitochondrial proteins in nuclear genomes of all former Archezoa, but the final proof came from experiments demonstrating the presence of MROs in these taxa 6. The Archezoa hypothesis was gradually replaced by a paradigm stating that mitochondria or mitochondrion-related organelles are present in all eukaryotes. The search for a truly amitochondriate eukaryote lost momentum.

The main diversity of MROs is hidden among microaerophilic and anaerobic microbial eukaryotes. Various eukaryotic lineages inhabit low oxygen environments and their mitochondria are pronouncedly reduced and lack most of the organellar proteins and functions, including membrane complexes functioning in oxidative phosphorylation. MROs known to date represent a spectrum of metabolic phenotypes at different levels of reduction: from hydrogen-producing mitochondria and hydrogenosomes producing ATP via substrate-level phosphorylation to mitosomes, which are not involved in ATP generation at all 7. Hydrogenosomes and mitosomes do not contain their own genomes and fully rely on proteins transported from the cytosol.

The observed spectrum of extant MROs apparently originated via stepwise reduction of ancestral mitochondria accompanied by loss or replacement of mitochondrial proteins and functions. All these variously shaped organelles should provide some benefit to the cell, otherwise there would not be a reason to maintain them. ATP generation clearly isn’t always such a reason as it is not produced in all MROs. It was widely believed that such a key and omnipresent function is the biosynthesis of Fe-S clusters via the mitochondrial ISC system 2. However, there are interesting examples demonstrating that under specific circumstances this function may be replaced or even moved outside the mitochondrion 8,9. Consequences of such functional rearrangements are remarkable, as will be discussed below.

## NEW SOLUTIONS FOR THE SYNTHESIS OF Fe-S CLUSTERS 

The ISC system in mitochondria and MROs assembles not only Fe-S proteins within the organelle, but also supplies an unknown essential sulphurous factor to the cytosolic Fe-S cluster assembly (CIA) machinery 10. Nevertheless, in three unrelated lineages of anaerobic microbial eukaryotes, the ISC pathway has been supplemented or replaced by a SUF (sulphur mobilisation) pathway acquired by horizontal gene transfer from Archea. In the stramenopile *Blastocystis*, the SufCB fusion protein was shown to function in the cytosol, while ISC is still present in the MRO 11. Likewise in *Stygiella incarcerata* (Excavata) the SufCB protein functions as an auxiliary machinery of the ISC system 12. In *Pygsuia biforma* (Breviatea), however, the SufCB protein is localized in the MRO and functionally replaces the ISC system 13, which is apparently absent. The SUF system is known to function in all plastids and in some prokaryotes, where it often serves as an accessory pathway to the ISC. Multiple independent acquisitions of the SUF system in eukaryotes without plastids suggest the functional benefit of this pathway. In prokaryotes the *suf* operon is up-regulated under oxygen stress and iron starvation, and it has been suggested that the SUF system in eukaryotes might provide a mechanism for the repair of oxygen-sensitive Fe-S proteins 11. It is unclear why in *P. biforma* the SUF system functionally replaced the ISC system, however, it was proposed that SUF system maintenance could have been favoured in its ancestors if they were periodically exposed to oxidative stress or iron starvation 13.

In Archamoebae we observe yet another pronounced modification of Fe-S cluster assembly, in which the ISC system was replaced by another analogous prokaryotic pathway called NIF (Nitrogen Fixation). *Mastigamoeba balamuthi *contains two sets of enzymes functioning in NIF, one is localized in the cytosol and the other in its MRO. The human parasite *Entamoeba histolytica *has only one version of these enzymes and it seems very likely that the whole synthesis of Fe-S clusters in *E. histolytica *takes place in the cytosol 14. Although the mitosome of *E. histolytica *runs neither Fe-S cluster synthesis nor generates ATP, it was still maintained in the course of evolution, supporting the generally accepted paradigm. The probable essential function of this particular mitosome is the production of specific sulphur compounds necessary for encystation of this parasite 15.

## A FLAGELLATE THAT CROSSED THE LINE

**Figure 1:**
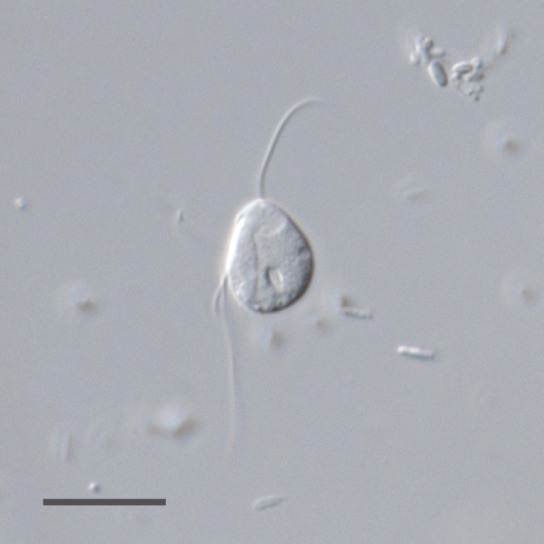
FIGURE 1: A living cell of *Monocercomonoides *sp. PA203 under differential interference contrast (DIC). Scale bar, 10 µm.

Our recent study 9 overturns the paradigm about omnipresence of mitochondria, which has been gradually strengthened during the last two decades by the investigation of more and more eukaryotes from low-oxygen environments. We chose the flagellate *Monocercomonoides* sp. (Fig. 1) for a detailed investigation, because available evidence has suggested a severe reduction of mitochondria in this lineage. *Monocercomonoides* is a genus of microaerophilic organisms living in the digestive tracts of animals. These microeukaryotes belong to Metamonada - a group exclusively consisting of anaerobes/microaerophiles typically possessing MROs. Notorious parasites, including diarrhea-causing *Giardia* (bearing mitosome), sexually transmitted *Trichomonas* and a fish parasite *Spironucleus *(bearing hydrogenosomes) are the best-known relatives of *Monocercomonoides.* No organelle resembling MRO was ever observed in *Monocercomonoides* cells under transmission electron microscope 16,17, but as mentioned above, MROs have often been overlooked.

We performed genome and transcriptome sequencing of *Monocercomonoides *sp.*,* allowing us detailed and careful analyses of its cellular features. The search for homologues of nuclear genome-encoded proteins did not recover any mitochondrial or MRO-related proteins, such as components of protein import machinery, mitochondrial metabolite transport proteins or the mitochondrial ISC Fe-S claster assembly system. We also failed to find MRO-associated proteins through searches focused on the presence of one of several possible mitochondrial signal sequences, such as conserved N-terminal and C-terminal targeting signals. At the same time, we were able to verify the presence of genes encoding hallmark proteins of the Golgi complex, the spliceosome and other typical eukaryotic systems using very similar bioinformatics approaches. This confirmed the validity of our results, pointing to the absence of mitochondria, and also suggested that amitochondrial *Monocercomonoides *sp. is in other respects a typical eukaryotic cell. Bioinformatic reconstruction of its metabolism implies that all ATP production occurs in the cytosol via substrate level phosphorylation.

Since the Fe-S proteins are essential for viability of all cells in all domains of life, the lack of a mitochondrial ISC system in *Monocercomonoides *sp. suggested its replacement by an alternative. Indeed, we identified in its genome genes for the SUF system, the pathway known from prokaryotes, plastids, and several isolated eukaryotic lineages like *P. biforma*. *Monocercomonoides *sp. contains the most complete SUF pathway of eukaryotic anaerobes/microaerophiles; it consists of fusion protein SufSU, and proteins SufB and SufC, and it is theoretically fully functional. Unlike *Pygsuia*, the SUF proteins of *Monocercomonoides *are localized in the cytosol as indicated by localization experiments in heterologous systems 9.

## THE SYNTHESIS OF Fe-S CLUSTERS DOES NOT NEED TO BE COMPARTMENTALIZED

The cytosolic localization of the complete Fe-S cluster assembly reported in *Monocercomonoides *and previously in *Entamoeba *is very rare among eukaryotes. The eukaryotic ISC system is always localized in mitochondria or MROs. When substituted by another pathway, the process often stays localized in the MROs, exemplified by SUF in *P. biforma *13 and NIF in *M*.* balamuthii *14. It was proposed that in eukaryotes, the reactions needed for the synthesis of Fe-S clusters, regardless of their evolutionary origin, demand compartmentalization 13. The two recent examples of *Monocercomonoides *and *Entamoeba*, however, demonstrate that this is not strictly true. We hypothesize that the reason for the mitochondrial localization of Fe-S cluster synthesis in almost all eukaryotes is the fact that it is needed for the biogenesis of Fe-S proteins in these compartments. The presence of an Fe-S cluster assembly machinery in MROs without any other Fe-S cluster-containing enzymes (e.g. in the mitosome of *Giardia*) is likely an evolutionary residuum - the lineage has not evolved an alternative solution on how to run this essential pathway. In two known cases, *Monocercomonoides* sp. and *E. histolytica*, the evolution happened to re-localize the process simultaneously with its horizontal gene transfer replacement by another prokaryotic pathway.

The scarcity of examples and unique combination of features in each described case, make it difficult to draw more general conclusions of evolutionary history of eukaryotes employing non-standard Fe-S cluster assembly machineries, particularly those localized outside MROs. The most useful data for the reconstruction of the evolution of MROs in *Monocercomonoides* and *Entamoeba *lineages will probably come from the investigation of their relatives. *Paratrimastix* *pyriformis*, a relative of *Monocercomonoides*, contains hydrogenosome-like organelles, whose function is not well understood, but which contain at least one biochemical pathway involved in amino acid metabolism 18. The existence of an MRO in *P*.* pyriformis *suggests that the absence of mitochondria in *Monocercomonoides *is due to a secondary loss and not the primitive state. Interestingly, *P*. *pyriformis* also lacks the ISC system, contains genes for the SUF system and the phylogenetic analyses suggest that these genes were in the common ancestor of *Monocercomonoides *and *Paratrimastix* 9. The localization of the SUF system proteins in *P*. *pyriformis *is unknown and revealing it may help to understand the loss of MRO in the *Monocercomonoides* lineage.

## LOSS OF ORGANELLE IS EXTREMELY RARE

Up until now, *Monocercomonoides *represents the only case of a eukaryote that has lost mitochondria. However, reductive evolution of plastids and mitochondria are in many aspects analogical and studies on plastid evolution might be useful to understand evolution of mitochondria. The reductive evolution of both organelles happened in a stepwise manner independently in many eukaryotic lineages and resulted in a range of remnant organelles with various metabolic properties. Retention of those reduced organelles is explained by the cellular dependence on the processes localized in them. There are only two well documented examples of plastid losses - in cryptosporidia 19 and in the parasitic dinoflagellate *Haematodinium* 20. The rarity of organelle loss highlights the difficulty of this evolutionary step. Those three known cases appear to have achieved organelle losses through minimizing the metabolic redundancy, although this redundancy might be eliminated in different ways: by reliance on host metabolism (*Cryptosporidium*), retention of cytosolic versions of the pathways (*Haematodinium*) or horizontal gene transfer resulting in relocation of the pathway to the cytosol as identified in *Monocercomonoides.* Together, those taxa show the manifold steps that are required and how unlikely it is to lose an organelle.
